# Associations of psychological factors, parental involvement, and adverse health behaviors with bullying among tunisian middle school students

**DOI:** 10.1186/s40359-023-01190-7

**Published:** 2023-05-12

**Authors:** Manel Ben Fredj, Cyrine Bennasrallah, Ines Amor, Faouzia Trimech, Hela Abroug, Imen Zemni, Wafa Dhouib, Meriem Kacem, Ines Bouanene, Asma Belguith Sriha

**Affiliations:** 1 Department of Epidemiology and Preventive Medicine, University Hospital Fattouma Bourguiba, Monastir, Tunisia; 2 Department of Community Medicine and Epidemiology, Faculty of Medicine of Monastir, University of Monastir, Monastir, Tunisia; 3 Research Laboratory “Technology and Medical Imaging”, Monastir, Tunisia; 4 Directorate of school and university medicine of Monastir, Monastir, Tunisia

**Keywords:** Bullying, Adolescent, Schools, Tunisia

## Abstract

**Background:**

Bullying is a serious problem that significantly affect adolescent well-being and health, needing the attention of teachers, school administrators, parents and public health professionals. In this study, we aimed at estimating the prevalence of bullying, from the perspective of victims in middle school students in the region of Monastir Tunisia, as well as analyzing its association with individual and family context variables.

**Methods:**

This is a cross-sectional study conducted in December 2017 and January 2018 among a sample of students from two middle schools in the region of Monastir (Tunisia), using the Global School-based Student Health Survey (GSHS) self-answered questionnaire. We defined bullying victimization as being bullied in at least one day in the previous 30 days. Binary logistic regression model was used to identify factors associated with being bullied.

**Results:**

Out of 802 students included in this study, nearly half (43.4%) reported having been bullied in the past month with CI _95%_: 38.9–48.2. Gender did not interact with this behavior: (44.5%; CI _95%_: 38.1–51.7) in boys versus (43.4% ; CI _95%_: 37.2–50.2) in girls. Univariate analysis indicated significant differences regarding some individual factors such as physical fight, cigarette smoking, feeling lonely and being worried, in terms of prevalence of being bully victims. There were no significant differences in parental factors between the two groups (being bullied or not). Multivariate analysis showed the following factors as independently associated with bullying: being involved in physical fight (OR = 2.4; CI_95%_:1.77–3.25), feeling lonely (OR = 3.38; CI_95%_ :2.04–5.57) and being worried (OR = 2.23; CI _95%_:1.44–3.43).

**Conclusion:**

Bullying victimization was common among school-going adolescents and was linked with physical fight and psychosocial distress. This study highlights the need for school-based violence prevention programs to address this problem among the students.

## Introduction

Adolescent violence is a major public health problem. It is the fifth leading cause of death in this age group, responsible for more than 12% of male deaths worldwide. It remains a growing, but a neglected issue globally [1]. According to the UNESCO, approximately 246 million children and adolescents face some form or another of school-based violence and harassment each year [2].

One of the forms of school violence is bullying, a type of peer violence considered as a public health problem. Bullying could include physical contact, verbal harassment, rumor spreading, intentionally social exclusion or lewd gestures. The act of bullying involves both a bully and a victim and it occurs repeatedly when there is a power imbalance between them [3].

Bullying is a global problem, common to many different countries and schools, with 20–56% of the world’s adolescents being involved every year in bullying situations [4–6]. Several studies have documented the adverse effects of bullying on children’s and adolescents’ developmental trajectories with elevated rates of anxiety, depression, and socio-emotional problems; behavioral difficulties, impaired academic performance, absenteeism and increased school dropout rates among victims of bullying [7–9].

Many individual and environmental factors may be related to the occurrence of this phenomenon. According to previous studies, bullying was linked with male gender, physical fighting, mental health disorders and risky behaviors such as substance use [10–14]. Other studies examined that relationship between parent–child communication and the risk of being bullied for a student [15]. This highlights how crucial it is to investigate further parental participation in this matter, especially in developing nations where parental involvement in bullying is still not well-researched.

In Tunisia, there have been few studies on bullying prevalence, which makes it difficult to establish trends over time. In this context, we conducted this research to focus on this phenomenon, estimate the nature and prevalence of bullying in Tunisian schools, monitor potential changes over time and pinpoint populations that may be more susceptible to bullying victimization.

Thus, this study aimed to estimate the prevalence of bullying, from the perspective of the victims in middle school students in the region of Monastir Tunisia, as well as to analyze its association with individual and family context variables.

## Methods

### Study design

This is a cross-sectional study conducted in middle schools belonging to the region of Monastir Tunisia in December 2017 and January 2018.

### Study sample

The sample size was calculated using a 95% confidence interval, 5% allowed error, a prevalence rate of bullying of 30.6% as reported previously [16], and a design effect of 2. The calculated sample size was 646. The sample was increased by 25% to account for nonresponse and recording errors. We used a two-stage cluster sampling design to recruit a representative sample of school students in grades 7–9 in Monastir governorate. Delegations were selected in the first stage and schools in the second stage. All students in randomly selected schools were eligible for inclusion in the study.

### Study instrument

The Global School-based Student Health Survey (GSHS) is a collaborative surveillance project designed to help countries measure and assess the behavioral risk and protective factors among students. It uses a self-answered questionnaire to obtain data on young people’s health behavior and protection factors related to the leading causes of morbidity and mortality among children and adults worldwide [17].

We used the Arabic version of GSHS questionnaire which is composed of 10 modules: demographics and anthropometry, dietary behaviors, tobacco use, hygiene, mental health, physical activity, protective factors, HIV knowledge, violence, and unintentional injuries. Modules of alcohol use and sexual behaviors were not addressed given the socio-cultural context.

### Studied variables

In the current study, we studied the questions related to bullying and the questions linked to some individual and parental factors. Two questions were included in the questionnaire to obtain information relevant to bullying; these were about how many days and how the student was bullied during the 30 days prior to the survey.

Concerning the dependent variable “being bullied”, it was interpreted from one question: “*During the past 30 days*, *how many days were you bullied*?”. The response options range was from “0 day”, “1or 2 days”, “from 3 to 5 days”, “from 6 to 9 days”, “from 10 to 19 days”, “from 20 to 29 days”, to “everyday”. For our analyses, participants were classified as bullying victim if they reported being bullied one day or more.

Furthermore, questions related to socio-demographic data, physical fight, smoking, students’ feelings and friendships, and parental engagement in student’s life were used for the assessment of associations [18]. See Table 1.

**Table 1:** Definition and coding of independent variables included in statistical analysis

Independent variable	Question in GSHS	Binary classification (No = 0/Yes = 1)
**Physical fight**	During the past 12 months, how many times were you in a physical fight*?”*	0 or 1 time: 0
		More than 2 times : 1
**Smoking**	During the past 30 days, on how many days did you smoke cigarettes?	0 days = 0
		At least 1 day = 1
**Loneliness**	During the past 12 months, how often have you felt lonely?	Never/Rarely/Sometimes = 0
		Most of the time /Always = 1
**Worries**	During the past 12 months, how often have you been so worried about something that you could not sleep at night?	Never/Rarely/Sometimes = 0
		Most of the time /Always = 1
**Close friends**	How many close friends do you have?	No friend: 0
		One friend or more: 1
**Missing classes**	During the past 30 days, on how many days did you miss classes or school without permission?	0 days: 0
		One day or more: 1
**Parental support**	During the past 30 days, how often did your parents or guardians understand your problems and worries?	Never/Rarely/Sometimes = 0
		Most of the time /Always = 1
**Parental supervision**	During the past 30 days, how often did your parents or guardians check to see if your homework was done?	Never/Rarely/Sometimes = 0
		Most of the time /Always = 1
**Food insecurity**	During the past 30 days, how often did you go hungry because there was not enough food in your home?	Never/Rarely/Sometimes = 0
		Most of the time /Always = 1

### Data collection and reporting procedures

The questionnaire was administered by the research team to all the students in the designated schools. All students who voluntarily participated, self-completed the questionnaires in their classrooms during a 1-hour class period. To ensure that they can honestly answer, they were informed of the confidentiality and anonymity of their responses. Moreover, teachers and.

administrators were not involved in the distribution of questionnaires neither while responding. In each classroom, two interviewers were present: one to read the questionnaire and the other to reply to any further explanation needed by students.

### Statistical analyses

Data collection and analysis were performed using the Statistical Package for Social Sciences (SPSS) version 21.0. Descriptive analysis involved an examination of the socio-demographic characteristics of the study sample (age and gender), the prevalence and the types of bullying, psychological factors (loneliness, being worried and having close friends), parental involvement factors (parental support, and parental supervision), adverse health behaviors (physical fights and smoking cigarettes), and other factors such as missing classes without permission and food insecurity).

Univariate analysis using the Chi-Square test was performed to estimate the significance of the association between being bullied and the formerly mentioned factors. Multivariate logistic regression analysis was performed in a further step to identify the independently associated factors with bully victimization. A p-value less than 0.05 was considered significant at 95% confidence level.

## Results

### Description of the study sample

A total of 802 schools students participated in the survey with a mean age of 13.40 ± 1.2 years. Among them, 47.8% (n = 382) were boys and 52.2% (n = 412) were girls. By school grades, 47.4% (n = 366) were in the 7th grade; 33.9% (n = 272) were in the 8th grade and 18.7% (n = 153) were in the 9th grade.

### Prevalence of bullying behavior

Nearly half of the study group (43.4% ; CI _95%_:38.9–48.2) reported having been bullied in the past month (44.5% among boys (CI _95%_:38.1–51.7) versus 43.4% among girls (CI _95%_: 37.2–50.2). Among the respondents, being made fun of with sexual jokes, comments or gestures was the predominant type of bullying (9.1%), followed by being kicked, pushed, shoved or locked indoors (3.6%). Moreover, being left out of activities and made fun of because of their body, were each reported by 3.5% of the victims. Being made fun of due to their religion and being made fun of regarding their race, nationality or color were less prevalent (2.5% and 1.9% respectively).

### Factors associated with being a victim of bullying

Among the study group, girls and boys were equally bullied (51.1% among girls vs. 48.9% among boys, p = 0.73). Physical fight and cigarette smoking were significantly more common among boys (66.9% and 81.1% respectively) than in girls (33.1% and 44.7% respectively) (Table 1).

For psychological factors, a significantly higher proportion of girls had been feeling lonely (67.7%, p = 0.001), worried (65.5%, p = 0.001), and had no close friends (68.4%, p = 0.03) compared with boys. Furthermore, boys reported missing classes more significantly than girls did (65.2% vs. 34.8%, p < 0.00), while there was no significant difference regarding parental support and supervision (Table 2).

**Table 2:** Characteristics of study population according to gender

Variable (N)	Total (%)	Boys n (%)	Girls n (%)	p-value
Being Bullied (791)				
Yes	348 (43.4)	170 (48.8)	178 (51.2)	0.73
No	443 (56.6)	211 (47.6)	232 (52.4)	
Age group (793)				
≤ 14 years	635 (80.1)	300 (47.2)	335 (52.8)	0.29
> 14 years	158 (19.9)	82 (51.9)	76 (48.1)	
Physical fight (794)				
Yes	441 (55.4)	295 (66.9)	146 (33.1)	< 0.000
No	353 (44.6)	87 (24.6)	266 (75.4)	
Cigarette smoking (794)				
Yes	74 (9.3)	60 (81.1)	14 (18.9)	< 0.000
No	720 (90.7)	322 (44.7)	398 (55.3)	
Loneliness (786)				
Yes	99 (12.7)	32 (32.3)	67 (67.7)	0.001
No	687 (87.3)	344 (50.1)	343 (49.9)	
Being worried (794)				
Yes	131 (16.6)	45 (34.4)	86 (65.5)	0.001
No	663 (83.4)	337 (50.8)	326 (49.2)	
Close friends (794)				
Yes	756 (95.3)	370 (48,9)	386 (51,1)	0.03
No	38 (4.7)	12 (31.6)	26 (68.4)	
Missing classes (794)				
Yes	146 (18.2)	92 (65.2)	49 (34.8)	< 0.000
No	656 (81.8)	290 (44.4)	363 (55.6)	
Parental Support (794)				
Yes	322 (40.5)	147 (45.7)	175 (54.3)	0.25
No	472 (59.5)	235 (49.8)	237 (50.2)	
Parental Supervision (789)				
Yes	464 (58.5)	217 (46.8)	247 (53.2)	0.3
No	325 (41.5)	164 (50.5)	161 (49.5)	
Food insecurity (786)				
Yes	59	25 (42.3)	34(57.7)	< 0.000
No	737	351 (48.2)	376(51.8)	

Table 3 depicts the results of the univariate-based analysis of the relationship between being bullied and the correlated factors. Adolescents with adverse health behaviors like fighting physically and consuming cigarettes reported being bullied more often than those in groups who did not. In relation to the mental health domain factors, adolescents who reported feeling lonely and worried were more likely to be bullied, while there was no such association with not having close friends. Additionally, we did not discover a connection between gender or age and missing class without permission. As for the parental involvement, parental support and parental supervision were not significantly associated with bullying victimization. Food insecurity was also associated with bullying victimization in univariate analysis.

**Table 3:** Factors associated with being bullied (results of univariate analysis)

Factors	Being bullied n (%)	p-value
Gender		
Male	170 (48.9)	0.73
Female	178 (51.1)	
Age group		
≤ 14 years	274 (43.1)	0.22
> 14 years	77 (48.4)	
Physical fight		
Yes	237 (53.6)	< 0.000
No	115 (32.3)	
Cigarette smoking		
Yes	41 (56.9)	0.02
No	311 (42.8)	
Loneliness		
Yes	75 (74.3)	< 0.000
No	272 (39.4)	
Being worried		
Yes	87 (66.9)	< 0.000
No	265 (39.7)	
Close friends		
Yes	331 (43.6)	0.15
No	21 (55.3)	
Missing classes		
Yes	68 (47.2)	0.4
No	284 (43.4)	
Parental Support		
Yes	139 (43.2)	0.65
No	213 (44.7)	
Parental Supervision		
Yes	192 (41.4)	0.1
No	155 (47.1)	
Food insecurity		
Yes	34 (57.0)	0.03
No	315 (43.1)	

Results of the multivariate logistic regression analysis showed that being involved in physical fight, feeling lonely and being worried remained associated with being bullied and were considered as independent associated factors. In fact, having been in a physical fight increased the odds of being harassed by 2.4-fold (p < 0.05; CI_95%_ [1.77–3.25]). Adolescents who suffered from loneliness were 3.38 times more bullied then those who did not (p < 0.05, CI_95%_ [2.04–5.57]). Feeling worried increased the odds of bullying victimization by 2.23-fold (p < 0.05, CI_95%_ [1.44–3.43]) **(**Table 4**).**

**Table 4:** Factors associated with being bullied (results of multivariate analysis)

Factors	OR	CI 95%		aOR	CI 95%	
		Inferior	Superior		Inferior	Superior
Physical Fight	2.90*	2.31	3.65	2.40*	0.77	3.25
Loneliness	3.96*	3.12	5.03	3.38*	2.04	5.57
Being worried	3.09*	2.46	3.90	2.23*	1.44	3.43

*Statistically significant (p < 0.000); OR = odds ratio; CI = confidence interval; aOR = adjusted odds ratio

## Discussion

Bullying among adolescents is a major problem in many developed and developing countries. Bullying victimization has adverse consequences on students’ overall health and school performances and should be addressed promptly. Existent research has demonstrated that victims of bullying have elevated rates of school absenteeism, lower academic achievement, adverse mental health (e.g. depression, anxiety, suicidal ideation), and physical health outcomes (e.g. somatic complaints, sleeping troubles, substance use and risky sexual behaviors) [19–23]. The victims were even proven to have long-term effects in their late adolescence and even adulthood [24–27].

In light of these facts, our study was conducted to estimate the prevalence of bullying and to explore the associated factors using an adapted Arabic version of an internationally recognized questionnaire (GSHS) [28].

In our study, nearly half of the study group (43.4%) reported having been bullied in the past month. After multivariate analysis, only the succeeding variables remained significant and were considered as independent associated factors: being involved in physical fight, feeling lonely, and being worried.

The present study findings demonstrate that the prevalence of bullying has increased by almost 10% (30.6%) when compared to the national GSHS held in 2008 [29]. However, another study conducted in Sousse governorate using the revised Olweus Bully/Victim Questionnaire reported that 16.7% of middle school students declared being bullied [30].

In comparison with countries from the Middle-East and North Africa, our current results regarding the prevalence of being bullied in middle schools were close to Algeria, Morocco, Yemen and Qatar results, while Egypt had a much more higher rates of students being bullied (70% ) [31]. In sub-Saharan Africa, the overall prevalence of bullying victimisation was 38.8% varying from 22 to 54.6% according to countries [6]. Research conducted in high-income countries showed that this phenomenon was less prevalent. A cross-sectional conducted in France in 2014 revealed that 13.4% of middle school adolescents were bully-victims [32]. Furthermore, results from the 2011 national Youth Risk Behavior Surveillance System in the United States indicated that 20.1% of students were bullied [4]. The difference in results might be explained by the increase in social violence since the 2011 Tunisian revolution. The resulted social and economic changes might have influenced such behavioural change, especially among vulnerable adolescent groups.

The current analysis provides support for many previous studies in showing that being involved in physical fight and mental distress (loneliness and anxiety) are associated factors for being bullied [12, 33–35]. These same factors have an interdependent relationship with bully victimization. They can be considered associated factors as we found in our study, or consequences. Similarly, other researchers have demonstrated that students who are bullied are more prone to antisocial behavior [3, 22, 36]. Therefore, they may be at an increased risk of being perpetrators of violence [18, 37, 38].

Our findings about the lack of age difference in reporting bullying victimization experience is consistent with other studies [39, 40]. Our current results regarding the absence of a relationship between gender and being bullied are comparable to past studies results [40], and contrasting with a large number of studies that have demonstrated that boys were more likely to be bullied [41, 42].

Adverse health behaviors such as cigarette smoking have been reported to be associated with being a bully-victim [40, 43]. Though this relationship was identified in our primary univariate analysis (56.9% among smokers vs. 42.8% among nonsmokers, p = 0.02), yet it was eliminated after logistic regression analysis. Smoking was significantly associated with being bullied in previous studies [44]. This could be attributed to a coping mechanism against stressful life experiences, such as bullying.

In our study, we did not find an association between parents’ support or supervision and being victim in school bullying. However, parental involvement in the adolescent’s life has been identified as a protective factor from being harassed by many researchers [29, 33]. Recent studies using GSHS in Oman and Vietnam identified parental involvement as a protective factor from bullying victimization [45, 46]. This might be explained by a healthy parent–child relationship, allowing a protective environment against the peer pressure influences [45, 47]. Indeed, modern trends highlighted adolescent’s needs for independence, but parental support plays a key role in guiding children to the next level of social skills and promoting their mental health [48].

Our findings could be valuable to our society in this particular time of transition following the revolution and call for effective strategies to address and prevent the short and long-term potential mental and physical health consequences of being of bully-victim.

Some limitations should be considered in the interpretation of our results. First, the current survey enrolled only public school- going adolescents who may not be representative of all adolescents in the region of Monastir since we did not include private schools. In addition, the questionnaire was self-completed; some study participants may have misreported either intentionally or inadvertently on any of the questions asked. Intentionally misreporting was probably minimized by the fact that students completed the questionnaires anonymously. Since the current study is cross-sectional, the temporal relationship of several associations could not be established. Future longitudinal research is needed to truly establish causality.

## Conclusion

Bullying is a serious public health problem among adolescents worldwide. This phenomenon requires the attention of school health authorities, education administrators and parents.

School health authorities should consider the effect of bullying victimization on the psychological and physical health of students. A multisectoral approach drawing on contributions from many domains should be adopted to confront the increasing frequency of this phenomenon. The role of policy makers is crucial to help school authorities in developing a system for safe and confidential recording, reporting and management of bullying and fighting at schools, and to create a safe and supportive environment for all [34].

Integration of anti-bullying and violence prevention education into the curriculum might be more effective to their prevention in the long term. The implementation of anti-violence school-based programs in Tunisia is a priority. Better awareness of bullying prevalence and its potential associated factors may lead to improvements in health promotion programmes and guide targeted interventions [33]. Promoting healthy behaviors and protecting youth from many health risks should be enforced to provide a next generation with a decreased frequency of chronic diseases in adulthood, and with a more capacity to product and develop.

## Data Availability

The datasets generated and/or analyzed during the current study are not publicly available due to reasons of patient confidentiality but are available from the corresponding author on reasonable request.

## Abbreviations

GSHS: Global School-based Student Health Survey
SPSS: Statistical Package for Social Sciences
OR: Odds ratio
CI: Confidence Interval
